# The Role of Intermittent Fasting in Parkinson's Disease

**DOI:** 10.3389/fneur.2021.682184

**Published:** 2021-06-01

**Authors:** Bryan J. Neth, Brent A. Bauer, Eduardo E. Benarroch, Rodolfo Savica

**Affiliations:** ^1^Departments of Neurology, Mayo Clinic, Rochester, MN, United States; ^2^Internal Medicine, Mayo Clinic, Rochester, MN, United States

**Keywords:** intermittent fasting, Parkinson's disease, mitochondrial dysfunction, oxidative stress, neuronal loss, alpha synuclein

## Introduction

Parkinson disease (PD) is the second most common neurodegenerative disease, affecting ~2% of the population over age 70. Disease prevalence increases with age and, given the aging population, may triple in the next few years (1). The neurodegenerative mechanism leading to PD is still not completely elucidated. Alpha-synuclein may drive the neurodegenerative process of PD. When aggregated in neurons as intracellular Lewy bodies, it constitutes the pathologic hallmark of PD (2). On the other hand, mitochondrial dysfunction, oxidative stress, and selective neuronal loss each contribute to PD pathology (3).

Unfortunately, there remains no disease-modifying treatment in PD despite multiple trials of promising preclinical targets. Supplements and dietary interventions have been periodically considered as possible therapeutic approaches to impact disease progression and severity in related neurodegenerative disorders (3). One such intervention is intermittent fasting (IF). This viewpoint seeks to describe the putative pathophysiologic relationships among mitochondria, alpha-synuclein and PD risk genes and to provide a background for the rationale or the use of IF and similar mitochondrial-targeting therapies in PD. Finally, we propose an outline for determining the efficacy of an IF intervention in PD.

## Pathologic Hallmarks of PD

### Alpha-Synuclein

Alpha-synuclein is a 140-amino acid protein existing in several forms and is believed to contribute to PD pathogenesis. In PD, Lewy bodies typically concentrate in dopaminergic neurons of the substantia nigra, with alpha-synuclein-containing neurites more evenly distributed throughout the brain (2, 4). The oligomeric form of alpha-synuclein has been associated with various forms of toxicity. Key observations suggest alpha-synuclein is toxic to mitochondria and may promote PD pathogenesis (2). Likely mechanisms of pathogenicity include increased reactive oxygen species (ROS) generation and oxidative stress (5), increased neuroinflammation (2), modified membrane permeability (6), disrupted intracellular calcium homeostasis (7), altered autophagy and ubiquitin-proteasome function (8), endoplasmic reticulum-related stress (9), cytochrome c release (10), disrupted mitochondrial dynamics and altered bioenergetics, which lead to reduced synaptic transmission and cell death (11).

### Mitochondrial and Bioenergetic Dysfunction

#### Overview

Energy production in the form of adenosine triphosphate (ATP) is crucial for the brain, which seems to use ~20–25% of glucose and oxygen of the entire body (12). Importantly, oxidative phosphorylation is the most ATP-producing mechanism of the brain and is dependent on mitochondrial respiration.

#### Hereditary Forms of PD

Although the clinical symptoms of early and late-onset PD appear similar, it is important to differentiate likely differences in underlying pathophysiology (4). Several gene mutations that confer increased risk for hereditary PD are related to mitochondrial function, further supporting a role of mitochondrial dysfunction in PD.

**Figure 1:**
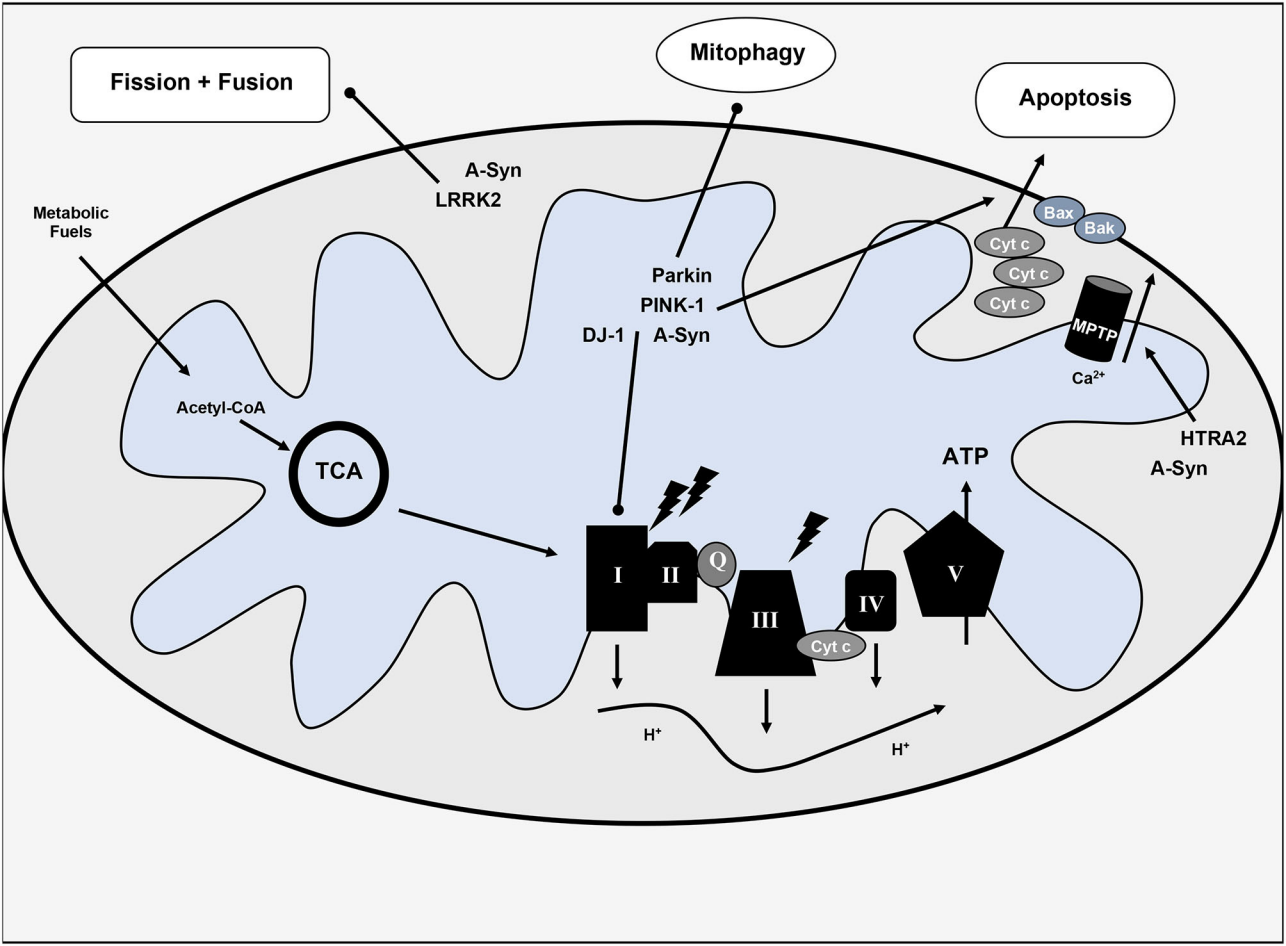
Overview of mitochondrial dysfunction in Parkinson's disease. Various hereditary forms of Parkinson's disease with implicated genes are included with their pathophysiologic mechanisms. TCA, tricarboxylic acid cycle or citric acid cycle; Cyt c, cytochrome c; Q, coenzyme Q; ATP, adenosine triphosphate; I-V, complexes I-V of the electron transport chain; MPTP, mitochondrial permeability transition pore; H^+^, protons; Ca2^+^, calcium, lighting bolt signifies oxidative stress.

Implicated genes include: alpha-synuclein (*SNCA* or PARK1), Parkin (PARK2), PINK-1 (PARK6), DJ-1 (PARK7), LRRK2 (PARKS8), and HTRA2 (PARK13) (2). Mutations in these genes either confer impaired mitochondrial function and/or increase of oxidative stress and inflammation resulting from this impairment. Notably, given the multifaceted role of mitochondria in cellular metabolism and cell death, several downstream sequelae may result from disrupted normal function. Mutations in alpha-synuclein, PINK-1, DJ-1, or LRRK2 may inhibit complex I of the ETC (2, 13). PINK-1 and Parkin mutations may also impair mitophagy and disrupt normal apoptotic pathways by interference with cytochrome c release and Bax translocation (14, 15). LRRK2 mutation affects mitochondrial fission; patients with this mutation have higher rates of mutation in mtDNA (16). HtrA2 alters the mitochondrial membrane potential and affects mitochondrial morphology (swelling) (17, 18). Additional gene mutations in UCHL-1, NURR1, and VPS35 correlate with PD and may also impact mitochondrial function. Bose and Beal (18) and Jin and Youle (19) offer a more complete review of the mitochondrial implications of PD-related genes. See Figure 1 for a visual representation of the impact of alpha-synuclein and PD-risk genes on mitochondrial function.

#### Metabolic Risk Factors for PD

Given the prevalence of vascular-metabolic disease and the rising incidence of neurodegenerative disorders, many epidemiologic studies have tried to define the link between systemic metabolic risk factors such as obesity and PD (20). Midlife triceps skinfold thickness was associated with a 3-fold increased risk to develop PD independent of other lifestyle and adiposity measures (21). Greater body mass index (BMI) has been related to increased PD risk, which was influenced by sex. On the other hand, other studies have shown no link between adiposity or BMI and PD risk (22). The link between type 2 diabetes mellitus and PD also remains controversial. Yet, it seems that insulin resistance or impaired insulin signaling may increase PD risk, linked to mitochondrial dysfunction (18, 23).

#### Consequences of Mitochondrial Dysfunction

**Figure 2:**
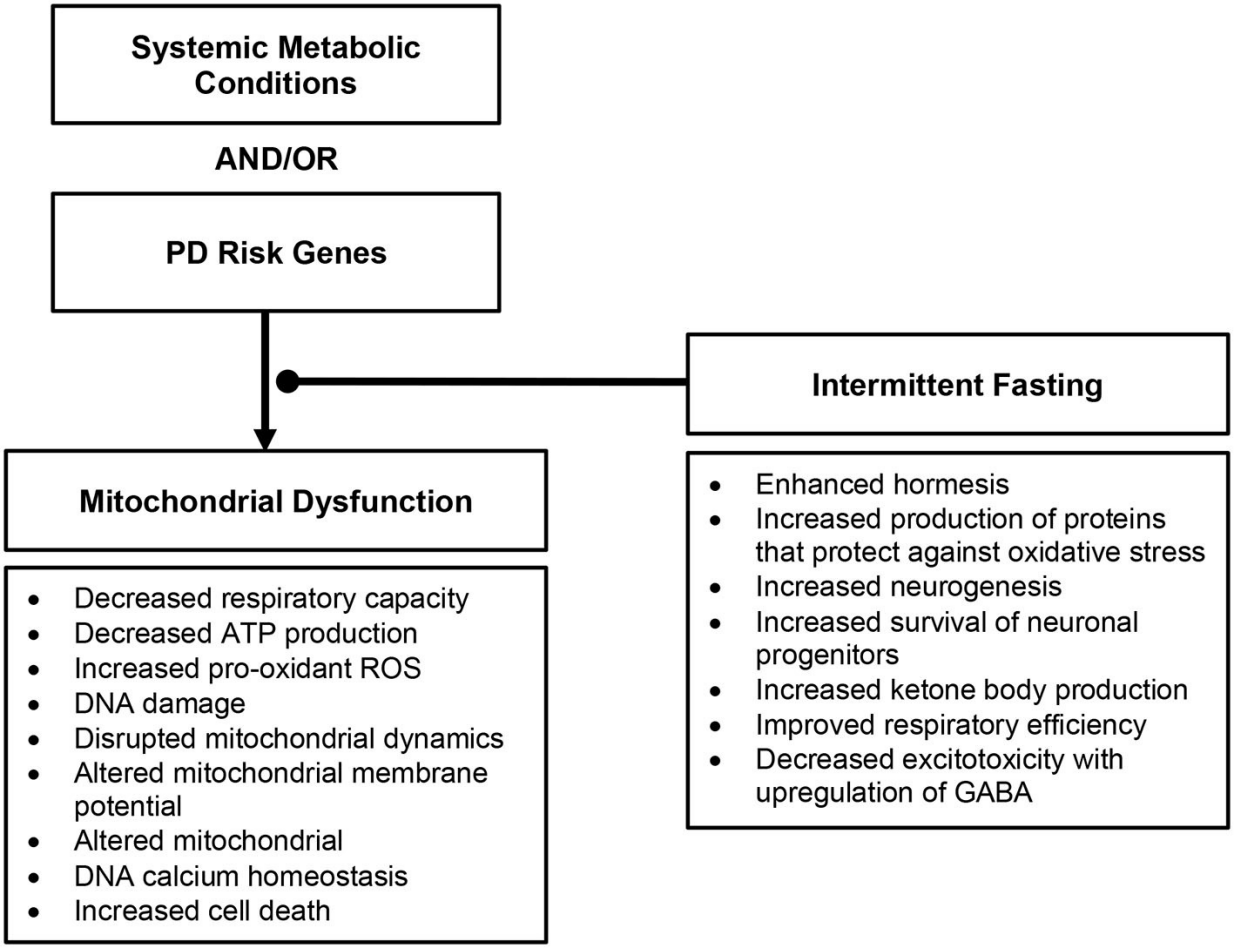
Overview of downstream effects of mitochondrial dysfunction in Parkinson's disease and the role intermittent fasting may play.

Many downstream effects of mitochondrial dysfunction have been associated to the development of PD (Figure 2), as reviewed in Rocha et al. (2); these include decreased respiratory capacity and bioenergetic function, increased production of pro-oxidant species and oxidative stress, disrupted mitochondrial dynamics, altered mitochondrial membrane potential, and calcium homeostasis – provoking further inflammatory response and cell death.

The ultimate sequelae of mitochondrial dysfunction and disrupted mitochondrial dynamics is cellular death, occurring in vulnerable cell populations precipitating the clinical phenotype of parkinsonism (24).The pathophysiologic hallmarks of mitochondrial dysfunction found in hereditary and sporadic PD offer important therapeutic potential to impact disease course.

## Overview of Therapies Targeting Mitochondrial Dysfunction

### Background

There is increasing focus on interventions targeting mitochondrial dysfunction in neurodegenerative disease (25). Many mitochondrial-targeted therapies are based on the concept of hormesis, defined as any process by which there is a biphasic response to increasing quantities and frequency of a given exposure (26). Hormesis is beneficial in conditions where smaller exposure leads to a beneficial or favorable response, and larger exposure leads to negative or toxic consequences (2). Mattson et al. review intermittent metabolic switching from glucose to ketones and back to glucose, through periods of short fasts and/or exercise (negative energy balance) and periods of eating and rest (positive energy balance) (27).

### Evidence in PD

#### Models

Current evidence regarding mitochondrial therapies in PD is from experimental models of disease. Anson et al. showed that C57BL/6 mice (susceptible to diet-induced obesity, diabetes mellitus, and atherosclerosis), maintained on an IF regimen consisting of fasting every other day, had improved insulin sensitivity (as evidenced by reduced serum glucose and insulin levels), increased BHB levels, and improved neuronal resistance to excitotoxicity, relative to mice on an *ad libitum* diet. These findings were independent of any caloric deficit or even weight loss hypothesized to contribute to the beneficial effect of fasting (28). Additionally, benefits of fasting are not limited to early therapeutic (pre-symptomatic) interventions. Elfawy and Das show improved motor and cognitive performance in rats that started an IF intervention (every other day feeding schedule) after 70% of their expected lifespan, later than many studies focusing on earlier intervention (29).

Both IF and CR have proven protective against neuronal excitotoxicity in rats and mice (27), potentially mediated through direct effects on mitochondrial function with inhibited mitochondrial permeability transition pore and retained mitochondrial calcium (27). A 6-month study of CR (70% baseline food intake) in a primate PD model led to better locomotor activity with higher striatal dopamine levels relative to *ad libitum*-fed controls (30).

Pharmacologic therapies targeting cellular metabolic and energetic activities also have been explored in PD. Oral anti-diabetic agents have been tested in animal models of PD. Metformin has been shown to have beneficial effects on PD-related pathology. Administration of metformin (3 weeks) led to improved motor activity, reduced oxidative stress and decreased neurodegeneration in a MPTP/probenecid mouse model of PD (31), potentially mediated through BDNF. Administration of metformin (5 weeks) led to decreased cell loss of dopaminergic neurons in the substantia nigra, increased dopamine levels, improved motor performance in a MPTP/probenecid mouse model of PD (32). Pioglitazone has been shown to be neuroprotective in MPTP mouse models of PD through peroxisome proliferator-activated receptor (PPAR) activation and inhibition of inducible nitric oxide synthase (iNOS) (33). Glucagon-like peptide 1 (GLP-1) has been targeted through GLP-1 agonist, Exenatide, showing neuroprotective effects (34). In a clinical study on PD patients, Exenatide provided benefit in off-medication scores on part 3 of the MDS-UPDRS at 60 weeks relative to controls with moderate PD (35). Unfortunately, despite that pharmacologic agents and supplements may provide therapeutic benefit, these are limited in targeting a solitary pathophysiologic mechanism, whereas dietary intervention that may have multiple targets.

#### Clinical

Few clinical studies have assessed the impact of dietary interventions or supplementation on mitochondrial dysfunction in PD. A 28-day ketogenic diet (4:1, fat:carbohydrates) trial in a limited number of PD patients (*n* = 7) showed varying adherence levels with two participants leaving the trials within the first week. However, patients completing the trial (mean decrease 43.4%) had an improved Unified Parkinson's Disease Rating Scale (UPDRS) score, with noted improvement in motor symptoms, mood, and energy level (36). Amigo et al. showed the feasibility of a dietary intervention aimed at ketosis in PD patients; 38/44 participants (86%) completed the 8-week study period: 18/22 randomized to the ketogenic diet and 20/22 randomized to the control low-fat/high-carbohydrate diet (37). Both groups showed improvement from baseline MDS-UPDRS Parts 1–4 scores; however, the KD group had the most significant improvement with greater changes in non-motor, daily-living experiences scores (UPDRS part I 4.58 vs. −0.99, *p* < 0.001) relative to the control group. These findings supported that KD also improved non-motor PD symptoms (37). Notwithstanding limited sample sizes and lacking confirmatory results, these trials highlight the feasibility of dietary intervention in PD.

## Proposed Intervention

### Why Use Intermittent Fasting in PD?

Without any available disease-modifying therapeutic options, one potential approach to slow progression of PD is to target key pathophysiologic changes in PD. Mitochondrial function is a possible target, especially in the PD cases when the mitochondria dysfunction seems to be the main and/or crucial mechanism. The benefits of IF likely originate from controlled amounts of small stress and recovery, or hormesis (38). In PD models, IF has resulted in improving insulin sensitivity (39), decreased excitotoxicity (40), reduced neurodegeneration (40), and protection against autonomic dysfunction (27, 41), and motor and cognitive decline (30). IF counteracts other pathologic features of PD by enhancing neurogenesis (37) and improving survival of neuronal progenitors (42). Moreover, the resulting ketosis may promote decreased excitotoxicity with the upregulation of GABA (43).

Importantly, IF may provide benefit for the non-motor symptoms of PD in addition to the motor symptoms. Griffioen and colleagues showed that intermittent fasting (energy restriction relative to a high energy diet) led to a decreased burden of alpha-synuclein in the brainstem that contributes to autonomic dysfunction (elevated resting heart rate, impaired cardiovascular stress response, reduced parasympathetic activity) commonly seen in PD (43). As autonomic dysfunction contributes to worse functional status, it remains an important therapeutic target in addition to motor symptoms (44, 45).

**Figure 3:**
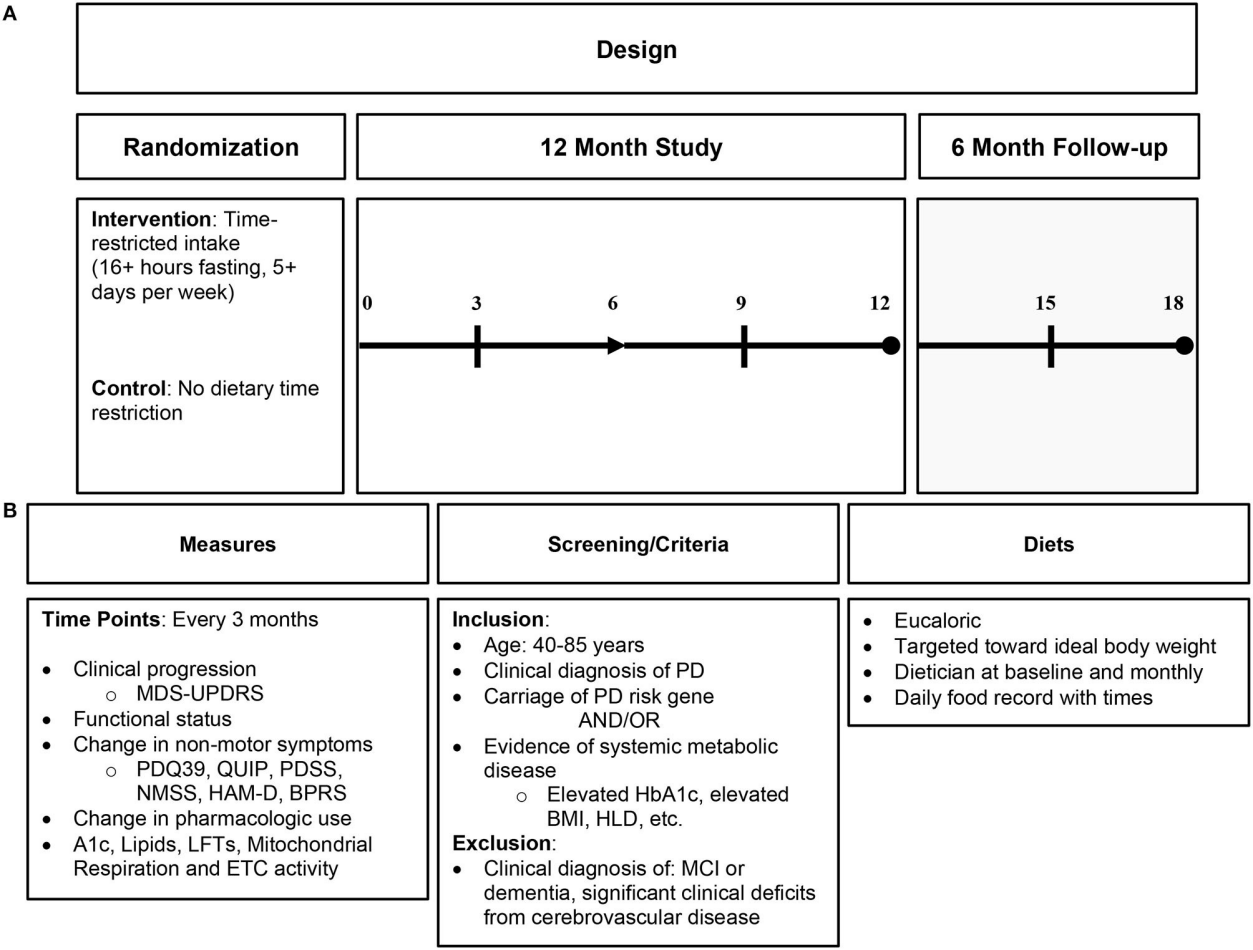
Proposed intermittent fasting intervention in Parkinson's disease. **(A)** Proposed study design; **(B)** proposed study details including measures of clinical endpoint with biomarkers, study criteria, and diet characteristics. PD, Parkinson's disease; HbA1c, hemoglobin A1c; HLD, hyperlipidemia; MCI, mild cognitive impairment; ETC, electron transport chain.

An IF dietary intervention is a more encompassing approach to mitochondrial dysfunction and its downstream consequences than nutritional supplementation and, thus, may be more beneficial to target similar pathology. IF likely influences several physiologic pathways, unlike supplementation, which only affects a narrower target (36). See Figure 3 for proposed dietary intervention.

### Is Intermittent Fasting Feasible in PD?

In theory, IF differs traditional restrictive diets (e.g., low fat or ketogenic diet). The only restriction in IF is a time-limited feeding interval. There is no limitation in foods to be consumed, although lean proteins, complex carbohydrates, and unsaturated fats are preferable. Importantly, the feeding phase of an IF diet is fundamental to the beneficial response (46). The fasting period may also vary. To improve adherence, fasting may be from 16 to 18 h daily or for a full 24-h period every other day with a normal eating pattern on opposite days. Because the diet does not restrict particular foods, there should be more consistent intake of quality foods and higher likelihood of long-term adherence, which is questionable with traditional restrictive diets. While ketosis is a sequela of IF, it is not the primary objective and thus is more amenable to dietary fluctuations.

Participants in any proposed trial should be carefully selected. An important question is whether IF may be more beneficial to those with sporadic PD or those with early-onset PD. As many genes implicated in early-onset PD relate to mitochondrial function, it seems logical that IF is useful in hereditary forms of PD. This may be a more readily targeted subset of PD patients. Nonetheless, mitochondrial dysfunction due to the presence of alpha-synuclein-related effects remains an important finding in sporadic PD, and IF should have beneficial effects in sporadic PD as well, particularly if there are metabolic risk factors or clear evidence for mitochondrial dysfunction in selection criteria for a given trial.

## Conclusion

PD is an incurable neurodegenerative disorder with hallmark pathologic features including alpha-synuclein accumulation, mitochondrial dysfunction, and oxidative stress. Targeting mitochondrial activity and oxidative stress may promote beneficial effects on PD. IF may positively impact the pathological mitochondrial changes seen in PD. It has minimal side effects and is less restrictive than other commonly used dietary interventions. Without disease-modifying treatment, we must continue to explore novel therapeutic approaches that target clinically relevant pathology. Lastly, IF may be an adjunctive treatment to counter pathophysiologic disease mechanisms and enhance therapeutic response.

## Author Contributions

BN: research conception and execution and writing first draft. BB: research organization and review and critique. EB: research execution and review and critique. RS: research conception, organization, and execution and review and critique. All authors contributed to the article and approved the submitted version.

## Conflict of Interest

BB receives support from the Department of Energy, Thorne Research, iPEx5 GmbH, and Reulay, Inc. RS receives support from the National Institute on Aging, the National Institute of Neurological Disorders and Stroke, the Parkinson's Disease Foundation, and Acadia Pharmaceuticals. The remaining authors declare that the research was conducted in the absence of any commercial or financial relationships that could be construed as a potential conflict of interest.
